# Impact of intra-partum azithromycin on carriage of group A streptococcus in the Gambia: a posthoc analysis of a double-blind randomized placebo-controlled trial

**DOI:** 10.1186/s12879-022-07080-4

**Published:** 2022-01-29

**Authors:** Isatou Jagne, Alexander J. Keeley, Abdoulie Bojang, Bully Camara, Edrissa Jallow, Elina Senghore, Claire Oluwalana, Saikou Y. Bah, Claire E. Turner, Abdul Karim Sesay, Umberto D’Alessandro, Christian Bottomley, Thushan I. de Silva, Anna Roca

**Affiliations:** 1 Disease Control and Elimination Theme, Medical Research Council Unit the Gambia at the London, School of Hygiene and Tropical Medicine, Banjul, The Gambia; 2 Department of Molecular Biology and Biotechnology, University of Sheffield, Sheffield, UK; 3 Department of Infectious Disease Epidemiology, London School of Hygiene and Tropical Medicine, London, UK

**Keywords:** Sub-Saharan Africa, Azithromycin, Bacterial carriage, Group A *streptococcus*, *Streptococcus dysgalactiae subspecies equisimilis*

## Abstract

**Background:**

Group A *Streptococcus* (GAS) is a major human pathogen and an important cause of maternal and neonatal sepsis. Asymptomatic bacterial colonization is considered a necessary step towards sepsis. Intra-partum azithromycin may reduce GAS carriage.

**Methods:**

A posthoc analysis of a double-blind, placebo-controlled randomized-trial was performed to determine the impact of 2 g oral dose of intra-partum azithromycin on maternal and neonatal GAS carriage and antibiotic resistance. Following screening, 829 mothers were randomized who delivered 843 babies. GAS was determined by obtaining samples from the maternal and newborn nasopharynx, maternal vaginal tract and breastmilk. Whole Genome Sequencing (WGS) of GAS isolates was performed using the Illumina Miseq platform.

**Results:**

GAS carriage was lower in the nasopharynx of both mothers and babies and breast milk among participants in the azithromycin arm. No differences in GAS carriage were found between groups in the vaginal tract. The occurrence of azithromycin-resistant GAS was similar in both arms, except for a higher prevalence in the vaginal tract among women in the azithromycin arm. WGS revealed all macrolide-resistant vaginal tract isolates from the azithromycin arm were *Streptococcus dysgalactiae* subspecies *equisimilis* expressing Lancefield group A carbohydrate (SDSE(A)) harbouring macrolide resistant genes *msr(D)* and *mef(A)*. Ten of the 45 GAS isolates (22.2%) were SDSE(A).

**Conclusions:**

Oral intra-partum azithromycin reduced GAS carriage among Gambian mothers and neonates however carriage in the maternal vaginal tract was not affected by the intervention due to azithromycin resistant SDSE(A). SDSE(A) resistance must be closely monitored to fully assess the public health impact of intrapartum azithromycin on GAS.

*Trial registration* ClinicalTrials.gov Identifier NCT01800942

**Supplementary Information:**

The online version contains supplementary material available at 10.1186/s12879-022-07080-4.

## Introduction

Pregnant women and neonates are at high risk of developing sepsis. In both groups, the risk persists for several weeks post delivery, and is associated with significant mortality [1, 2]. Morbidity due to maternal and neonatal sepsis is particularly high in sub-Saharan Africa (SSA) [3].

Globally, *Staphylococcus aureus* and Group B Streptococcus (GBS) are the main causes of maternal and neonatal sepsis [4–6]. However, Group A Streptococcus (GAS; *Streptococcus pyogenes*) is increasingly recognized as an important Gram-positive pathogen associated with maternal and neonatal sepsis [7–9]. GAS can cause both early and late onset of neonatal sepsis [10, 11], usually as a result of infection acquired through the birth canal [11]. GAS also causes non-invasive disease, including tonsillo-pharyngitis, skin infections and rheumatic fever that can result in rheumatic heart disease [12–14].

In high-income countries, it is estimated that the annual incidence of GAS-related maternal sepsis is 6 per 100,000 live births, with a 3.5% case-fatality ratio for invasive disease [15], and the incidence of GAS neonatal sepsis is 1.5 per 100,000 person years [16]. There is limited data on the burden of GAS infections in SSA due to the lack of systematic surveillance [17]. In the Eastern Cape, South Africa, the mean annual incidence rate of invasive GAS infection was 6 cases per 100,000-person years in all age groups (58% of samples from 18 to 64 year olds) [12]. In Kenya, the incidence of neonatal GAS sepsis was 0.6 cases per 1000 live births [18].

GAS colonizes the posterior pharynx and or skin of asymptomatic individuals who, although can transmit the bacterium, are less likely to transmit it than those with an acute GAS infection [19]. Understanding antibiotic resistance of GAS colonization is an indirect measure of understanding resistance of GAS causing acute infection in the community. In addition, there have been reports of an increased risk of neonatal infections associated to maternal vaginal carriage of GAS in the early postpartum period often with poor outcomes for these infants [20].

Here we present a posthoc analysis of the PregnAnZI trial [21] to determine the effect of 2 g intra-partum azithromycin on prevalence and antibiotic resistance of GAS in mothers and their newborns during the 4 weeks following prophylactic treatment. Whole genome sequencing (WGS) was done to further characterise the GAS isolates and perform phylogenetic analysis to explore the differences in the effect of azithromycin between anatomical sites.

## Methods

### Study design/population

This study is a posthoc (not pre-specified) analysis of data from a double-blind, placebo-controlled randomized trial in which women in labour were randomized to receive either a single dose of 2 g of oral azithromycin or placebo (ratio 1:1) [22]. The trial was conducted at the then-Jammeh Foundation for Peace (JFP) hospital, a government-run health facility located in western Gambia that manages approximately 4500 deliveries per year. The population in the catchment area is representative of The Gambia and covers its main ethnic groups [21]. Women who attended the JFP labour ward between April 2013 and April 2014, aged 18–45 years with no acute or chronic conditions were recruited into the trial. Details of exclusion criteria have been reported elsewhere [22]. The women had provided written informed consent to participate in the trial during previous antenatal care visits. Specifically, consent was obtained for the intervention, follow up, the collection of biological samples and the use of these samples in future unspecified analyses. The intervention was administered during labour, when the women presented to the health facility prior to delivery. Women and their newborns were followed for up to 8 weeks postpartum and biological samples were collected during the first 4 weeks [21, 22].

### Study samples

A nasopharyngeal swab (NPS) and a low vaginal swab (VS) were collected from women before the intervention was administered and during labour. Post-intervention samples included: (i) newborn NPS within 6 h after birth; (ii) samples collected during home visits at days 3, 6, 14 and 28 (NPS from mothers and newborns, and breast milk (BM) from mothers) and (iii) a VS collected in the health facility during the postnatal check at day 8–10 post-delivery [21].

### Sample collection

NPS were collected by passing the tip of a calcium alginate (Expotech USA Inc) swab across the mucosa of the posterior wall of the nasopharynx. The swab was rotated and left in the nasopharynx for approximately 5 s. The inoculated swab was placed immediately into a vial containing skim milk-tryptone-glucose-glycerol (STGG) transport medium and then into a cold box before being taken to the Medical Research Council Unit The Gambia (MRC) at the London School of Hygiene and Tropical Medicine (LSHTM) laboratories within 8 h of collection [22].

VS were collected by inserting a sterile cotton swab (Sterilin Ltd, UK) 2–3 cm into the vagina and rotating the swab with a circular motion, leaving it in the vagina for approximately 5 s. The inoculated swabs were then placed immediately into the vials containing STGG and put in a cold box before being transferred to the MRC laboratories within 8 h [22].

Breast milk samples were collected by first disinfecting the nipple and areola of the breast using sterile cotton soaked with 0.02% chlorhexidine. Mothers were then asked to manually express their milk. The first 0.5 mL was discarded. The following 1–2 mL was collected in a sterile plastic bijoux bottle put in a cold box and transferred to the MRC laboratories within 8 h [22].

All samples were stored at − 70 °C for subsequent processing in batches. The length of storage prior to processing differed between batches.

### Laboratory procedures

#### GAS culture from NPS, VS and breast milk samples

Samples were vortexed for 20 s prior to storage at − 70 °C for subsequent processing in batches. During processing, samples were allowed to thaw on ice. Each vial was then vortexed briefly in order to homogenise the medium and 50 μl was dispensed onto crystal violet blood agar (CVBA) (CM0085 Oxoid, UK + 0.02% crystal violet) for selective isolation of beta-haemolytic streptococci [22].

After 20–24 h incubation, presumptive beta-haemolytic colonies were streaked onto blood agar to obtain a pure growth. A catalase test was performed to differentiate the presumptive streptococci from staphylococci. Beta-haemolytic and catalase**-**negative isolates were grouped using the Streptex grouping kit (Remel R30950501) and ultimately reported as group A, B, C, D, F or G [22].

#### Antimicrobial susceptibility testing

Pure morphologically similar colonies were made into a suspension equal to 0.5% MacFarland’s standard and streaked evenly over the surface of Muller Hinton Agar (MHA). Antimicrobial resistance was evaluated using the disk diffusion method (15 ug azithromycin disk) and all resistant isolates (zones of inhibition ≤ 13 mm) were confirmed using E-test (AZ 256, range 0.016–256 mg/L, Biomerieux) [22]. The CLSI 2016 guidelines were used to interpret azithromycin susceptibility results.

#### Whole genome sequencing

Extracted DNA from all GAS isolates (isolated at any timepoint) was used for WGS by Illumina Miseq. Paired end reads were quality checked (FastQC) and trimmed for adaptor contaminant and low Q-score bases (Trimmomatic), this was followed by de novo assembly using k-mer settings of 21, 22, 55 and 77 (SPAdes) [23, 24]. Assembled genomes were checked for post-assembly quality (QUAST) [25]. Criteria for inclusion in further analysis were < 500 contigs, 1.6–2.0 Mb (*S. pyogenes*) or 2.0–2.4 Mb (*Streptococcus dysgalactiae* subspecies *equisimilis*; SDSE) assembly length, and > 90% reference genome coverage (*S. pyogenes*). Assembled genomes were annotated using Prokka and the core genome determined using Roary [23, 24]. Antimicrobial resistance genes were identified using ABRicate with the Resfinder database [25, 26]. *Emm*-typing was performed using the bioinformatics method described at https://github.com/BenJamesMetcalf and the CDC *emm*-typing database (https://www2.cdc.gov/vaccines/biotech/strepblast.asp). Core genome alignments were used to draw maximum likelihood phylogenetic trees (RAxML) with 1000 bootstraps [26]. For comparative phylogenetic analysis of SDSE isolates, complete genomes were obtained from NCBI Genome resource (https://ncbi.nlm.nih.gov/genome/genomes/823?) and from a previous study reporting SDSE expressing Lancefield group A (SDSE(A)) [27]. Phylogenetic trees were visualized using iTOL (https://itol.embl.de/). All sequence data are publicly available at the European Nucleotide Archive (Project Accession PRJEB36490).

### Data management and statistical analysis

The data were double entered into Open Clinica and analysed using STATA 16. Only participants with complete data at all timepoints were included in the analysis. An individual was considered positive for GAS carriage if GAS was present at any time point after treatment (3, 6, 14 or 28 days for NPS and breastmilk samples and 8–10 days for VS). The proportion positive was compared between arms using risk ratios (RR) and Fisher’s exact test was used to obtain a p-value for this comparison. P-values were not adjusted for multiple comparisons [28]. The proportion of resistant individuals was analysed similarly. In the determination of resistance, we considered an individual resistant at an anatomical site if any isolate from the site was resistant.

## Results

### Study population

**Fig. 1:**
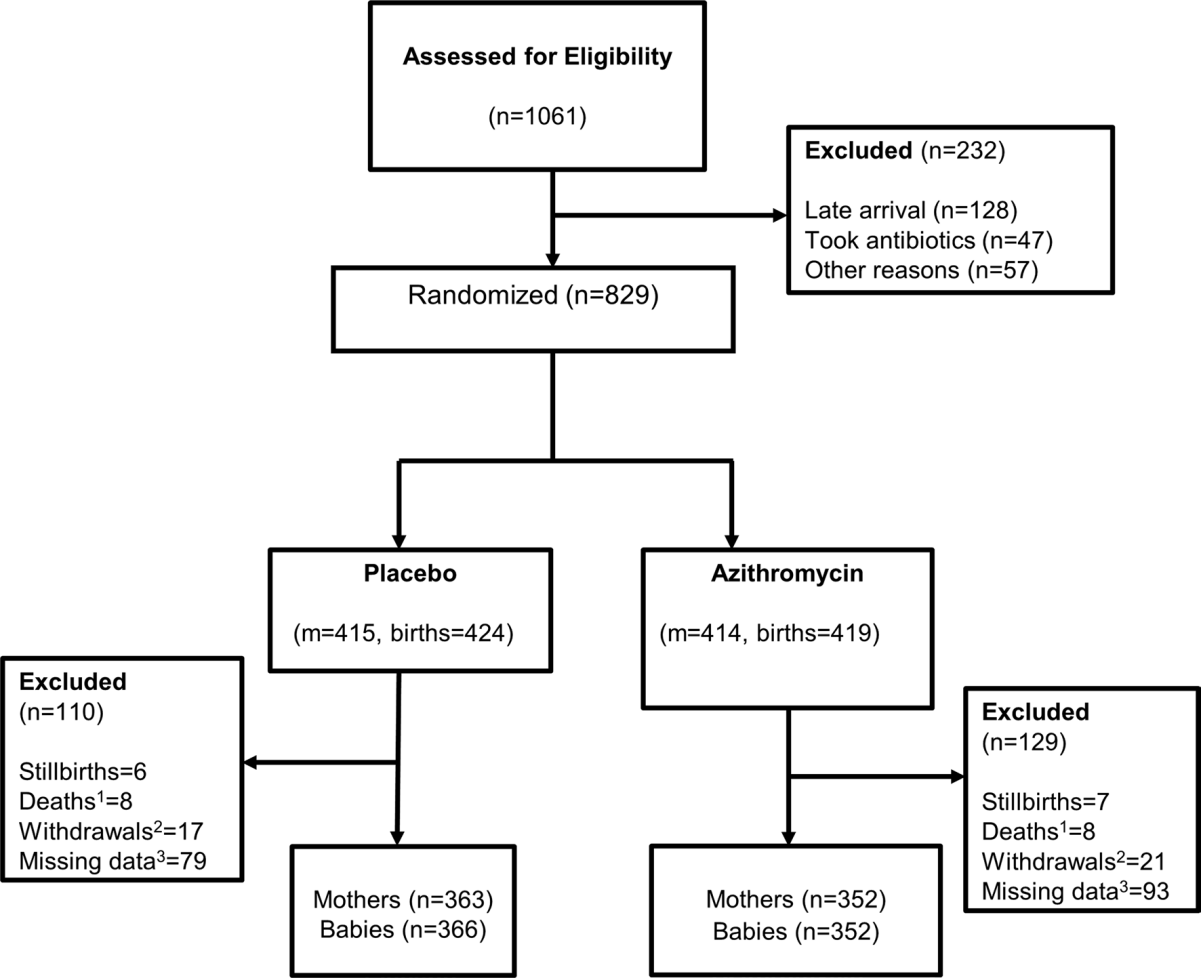
Trial Profile. ^1^All deaths were child deaths, there were no maternal deaths in the trial. ^2^All withdrawals involved a mother-pair (including twins). ^3^Mother/baby pair with ≥ 1 missing sample

A total of 829 women were recruited (414 women in the azithromycin and 415 in the placebo arms) and delivered a total of 843 babies, including 13 stillbirths. Overall, 715 mother-newborn pairs (86.2%) had all study samples collected and are part of this posthoc analysis (Fig. 1). Most women were 20–29 years old and the major ethnic group was Mandinka. Approximately 66.6% of deliveries occurred during the dry season (November to May), 5.0% of the newborns were low-birth weight and slightly more than half were males (Table 1).

**Table 1:** Baseline demographic characteristics of study participants

Characteristics	Placebo n (%)	Azithromycin n (%)
Mothers	n = 363	n = 352
Age		
18–19 years	32 (8.8)	20 (5.7)
20–29 years	239 (65.8)	229 (65.1)
≥ 30 years	92 (25.3)	103 (29.3)
Ethnicity^a^		
Mandinka	160 (44.1)	140 (39.8)
Wolof	42 (11.6)	42 (11.8)
Jola	53 (14.6)	62 (17.6)
Fula	57 (15.7)	62 (17.6)
Other	51 (14.1)	43 (12.2)
Season of delivery		
Dry (Nov–May)	244 (67.2)	232 (65.9)
Rainy (June–Oct)	119 (32.8)	120 (34.1)

Newborns	n = 366	n = 352
Birth weight^b^		
Low birth weight (< 2.5 kg)	23 (6.3)	13 (3.7)
Normal birth weight (> 2.5 kg)	338 (93.7)	338 (96.3)
Apgar score^c^ (at birth)		
0	0	0
1–6	3 (0.8)	1 (0.3)
7–10	362 (98.9)	350 (99.4)
Sex of child		
Male	195 (53.3)	180 (51.1)
Female	171 (46.7)	172 (48.9)

^a^Ethnicity missing in n = 3, ^b^Birth weight missing in n = 6, ^c^Apgar score missing in n = 2

### GAS carriage

Overall, 30 women and 9 newborns had at least 1 sample positive for GAS; 7 women and one baby had 2 positive samples and one woman had 5 positive samples (total of 51 GAS isolates).

#### Study mothers

**Fig. 2:**
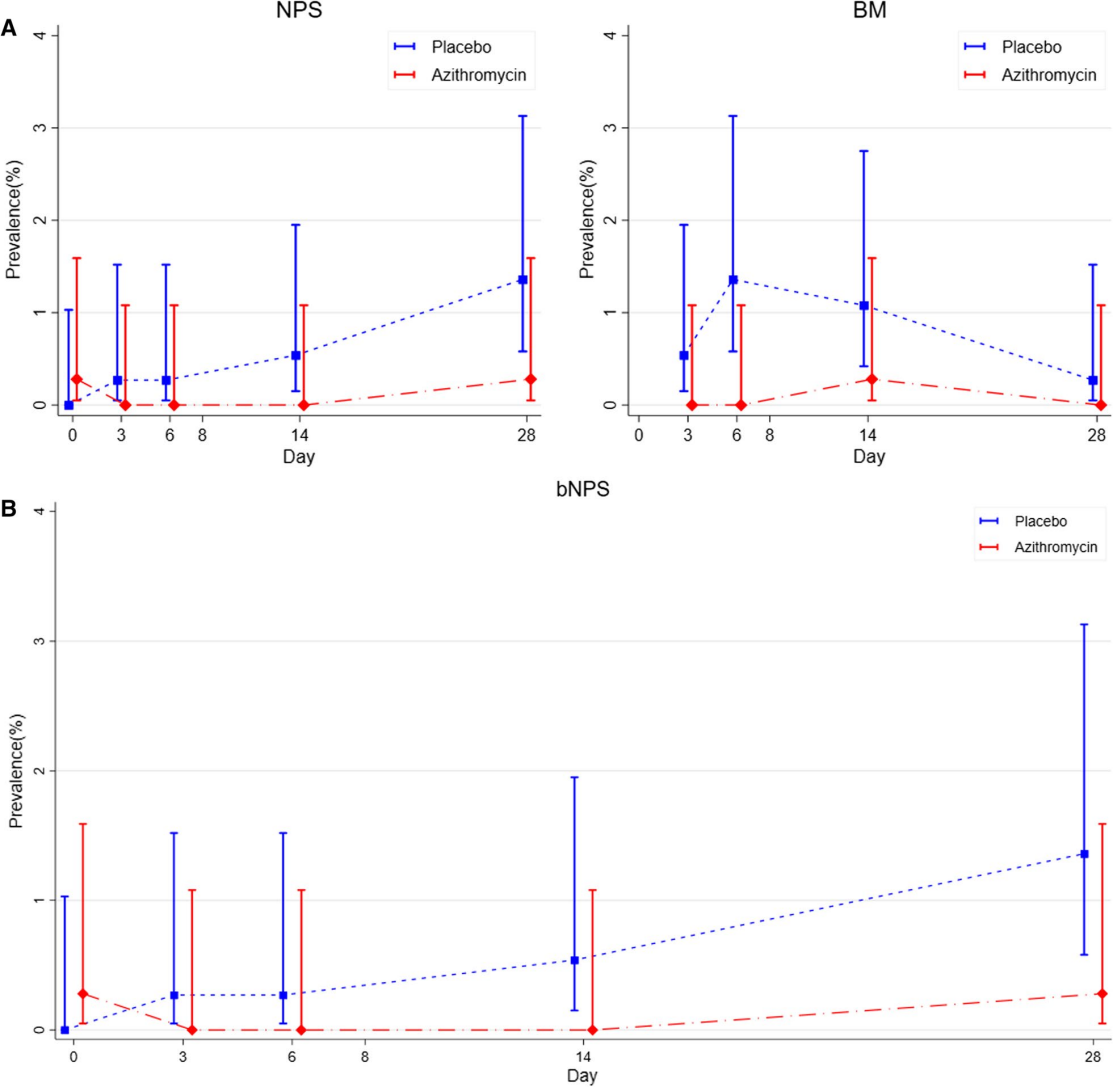
Maternal and neonatal carriage of GAS at different body sites and timepoints. **A** i. maternal nasopharyngeal carriage and ii. Breastmilk carriage of GAS at days 0, 3, 6, 14 and 28 post-delivery in the azithromycin and placebo arms. **B** Neonatal nasopharyngeal carriage of GAS at days 0, 3, 6, 14 and 28 after birth in the azithromycin and placebo arms

Pre-intervention (day 0) GAS carriage was uncommon and similar in the two study arms, both in the nasopharynx and in the vaginal tract (Table 2, Fig. 2A). Post intervention azithromycin reduced GAS carriage in the nasopharynx (0.28% versus 1.93%, p = 0.069) and breast milk (0.28% versus 2.48%, p = 0.021) but not in the vaginal tract (1.99% versus 1.93%, p = 1.000) (Table 2).

**Table 2:** Prevalence of GAS carriage in the nasopharynx, breastmilk and vaginal samples of mothers and nasopharynx of babies

Mothers				
	Placebo (%) n = 363	Azithromycin (%) n = 352	Risk ratio (95% CI)	p value
Nasopharyngeal carriage				
Pre-intervention^a^	0	1 (0.28)	–	0.492
Post iIntervention^b^	7 (1.93)	1 (0.28)	0.15 (0.02, 1.19)	0.069
Breastmilk carriage^c^				
Post intervention^b^	9 (2.48)	1 (0.28)	0.11 (0.01, 0.90)	0.021
Vaginal carriage				
Pre-intervention^a^	1 (0.28)	2 (0.57)	2.06 (0.18, 22.64)	0.619
Post iIntervention^d^	7 (1.93)	7 (1.99)	1.03 (0.37, 2.91)	1.000

Newborns				
	Placebo (%) n = 366	Azithromycin (%) n = 352	Risk ratio (95% CI)	p value
Post intervention^b^	7 (1.91)	2 (0.57)	0.30 (0.06, 1.42)	0.178

^a^Carriage before treatment (day 0)

^b^Carriage on one or more days after treatment (day 3, 6, 14 or 28)

^c^There are no pre-intervention samples for the breast milk

^d^Day10 (day 8–13)

#### Study newborns

GAS carriage was also uncommon in the newborns. Although there were fewer cases of carriage in the azithromycin arm, the difference was not statistically significant (0.57% versus 1.91%, p = 0.178) (Table 2, Fig. 2B).

### Azithromycin resistance

#### Study mothers

The occurrence of azithromycin-resistant GAS was similar between study arms for all sample types except for vaginal samples where resistant isolates occurred more frequently in the intervention arm [1.99% vs. 0.28%, p = 0.035] (Table 3).

**Table 3:** Carriage of resistant GAS in the nasopharynx, breastmilk and vaginal samples of mothers and nasopharynx of babies

Mothers				
	Placebo (%) n = 363	Azithromycin (%) n = 352	Risk ratio (95% CI)	p value
Nasopharyngeal carriage				
Pre-intervention^a^	0	0	–	–
Post-intervention^b^	2 (0.55)	1 (0.28)	0.52 (0.05, 5.66)	1.000
Breastmilk carriage^c^				
Post-intervention^b^	0	1 (0.28)	–	0.492
Vaginal carriage				
Pre-intervention^a^	0	1 (0.28)	–	–
Post-intervention^d^	1 (0.28)	7 (1.99)	7.24 (0.87, 56.92)	0.035

Newborns				
	Placebo (%) n = 366	Azithromycin (%) n = 352	Risk ratio (95% CI)	p value
Post-intervention^b^	1 (0.27)	1 (0.28)	1.04 (0.07, 16.56)	1.000

^a^Carriage before treatment (day 0)

^b^Carriage on one or more days after treatment (day 3, 6, 14 or 28)

^c^ There are no pre-intervention samples for the breast milk

^d^Day 10(day 8–13)

#### Study newborns

Only two resistant isolates were identified in newborns; one in each arm. (Table 3).

### Whole Genome Sequencing

**Fig. 3:**
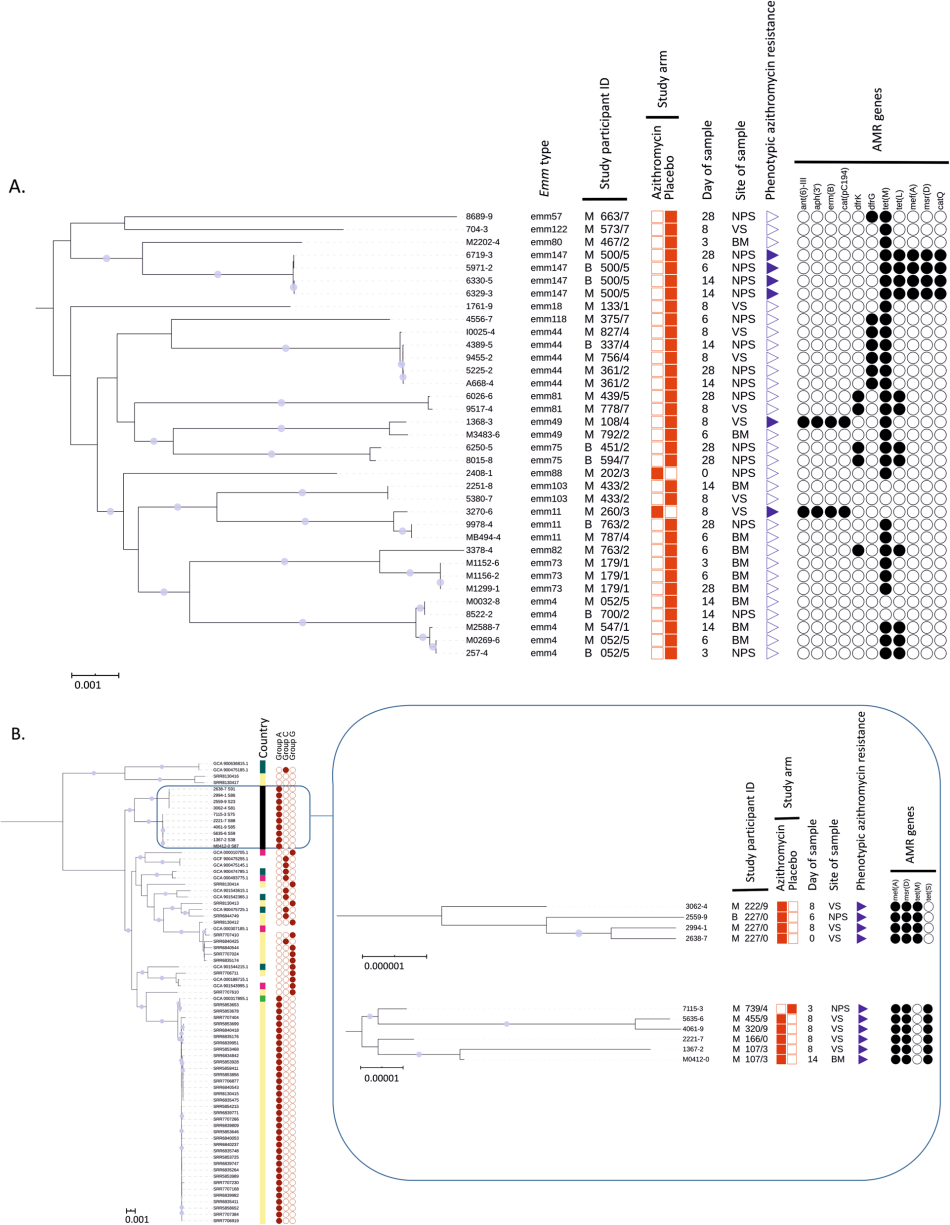
Midpoint rooted maximum likelihood core-genome phylogenetic analysis using RAxML GTRCAT model with 1000 bootstrap replicates. Circle symbols indicate > 99% bootstrap support. **A** Core-genome (1299 genes) phylogenetic analysis of 35 *S. pyogenes* isolates from the study cohort. **B** International contextualization (based on core genome of 1221 genes) of 10 *S. dysgalactiae* subspecies *equisimilis* isolates with individual core-genome (upper clade: 2106 genes, lower clade: 2078 genes) phylogenetic analysis of the two distinct clades in the study cohort. (Annotation key: country of origin; black = the Gambia, yellow = USA, dark green = UK, light green = Germany, pink = Japan, white = unknown, symbols; filled = present, unfilled = absent, no symbol = unknown; study participant ID (unique study identifier for mother/baby units); M = mother, B = newborn, NPS = nasopharyngeal swab, VS = vaginal swab, BM = breast milk, AMR = antimicrobial resistance)

Of the 51 GAS isolates, one sample was not retrieved, and five samples (isolated from four mothers) had poor sequences and were excluded from the WGS analysis. Of the remaining 45 isolates (Additional file 1), WGS confirmed that 35 were *S. pyogenes* (2 from the azithromycin arm and 33 from the placebo arm of the trial), from which we detected 16 *emm* types. The most common were *emm*4 and *emm*44 (5 isolates each, 14.2%) and *emm*147 (4 isolates, 11.4%). The remaining 10 isolates were SDSE. All were phenotypically resistant to azithromycin and 9 were from participants in the azithromycin arm. All 10 SDSE(A) isolates were retested with Streptex grouping kit and were confirmed as Lancefield group A beta-haemolytic streptococci. We observed that the most common resistance mechanism was by efflux with 14 out of 16 azithromycin-resistant isolates (including all SDSE(A)) harbouring both mefA and msrD genes (mefA-msrD). In *S. pyogenes*, mefA and msrD genes were adjacent to each other and located five genes upstream of catQ on what appeared to be a phage-like mobile genetic element, integrated downstream of rlmD (23 s rRNA methyltransferase). In SDSE(A), mefA and msrD were also present on a mobile genetic element that showed some similarity to that found in the reference SDSE strain AC-2713 (HE858529.1) integrated between comEC and comEA but differed in gene content between the two lineages of SDSE(A). The complete sequences of the mobile elements for both *S. pyogenes* and SDSE(A) could not be determined due to contig breaks in the de novo assemblies. *S. pyogenes* isolates recovered from the same mother from different biological sites or study timepoints, as well as isolates from their new-borns, all clustered together and belonged to the same *emm* type (Fig. 3A). Similar phylogenetic and epidemiological concordance was seen in the SDSE(A) isolates, including a neonatal NPS isolate closely linked to those recovered from the baby’s mother (Fig. 3B).

## Discussion

One oral dose (2 g) of azithromycin given to women in labour reduced occurrence of GAS carriage among women and their babies in the nasopharynx and breast milk without an increase of azithromycin resistance in isolates in these sample sites. In contrast, the intervention did not have any effect on the occurrence of GAS carriage in the vaginal tract but induced an increase in the carriage occurrence of azithromycin resistant SDSE(A).

Previous results from this study have shown that a single oral dose (2 g) of azithromycin given to women in labour reduced the prevalence of *S. aureus, S. pneumoniae* and GBS carriage in the mother (nasopharynx, breast milk and vaginal tract) and the baby (nasopharynx) [21]. The current analysis shows that the intervention also reduced the prevalence of GAS carriage in the breast milk and nasopharynx of study women and, less clearly, in the nasopharynx of their newborns. Despite substantial reduction of GAS carriage, we did not observe any short-term increase of azithromycin resistance in these two anatomical sites.

Conversely, the intervention did not have any effect on the prevalence of GAS carriage in the maternal vaginal tract but induced an increase in azithromycin resistance. WGS revealed that GAS vaginal carriage in the azithromycin arm was primarily due to azithromycin-resistant SDSE(A), whereas in the placebo arm, GAS vaginal carriage was entirely due to azithromycin-susceptible *S. pyogenes*. In our previous analysis on *S. aureus*, lower reduction in carriage in the vaginal tract alongside a higher prevalence of resistance was also observed [21]. It is not clear why the effect of the intervention in the vaginal tract differs from other body sites. The concentration of azithromycin in the vaginal tract may be lower and fall more rapidly than in other anatomical sites. We had previously shown a very high concentration of azithromycin in breast milk during the 4 weeks following the intervention, with a peak during the first 6 days (concentration > 4000 µg/L) [29]. A different study using a single dose of azithromycin (1 g) showed the azithromycin concentration in the vaginal tract was much lower than the breast milk concentration we observed [30]. In this study, the peak concentration occurred during the first 24–48 h following the intervention [30], long before the post-intervention VS were collected in our study. It is possible that removal of *S. pyogenes* from the vaginal tract allows azithromycin-resistant SDSE to thrive, whereas in other anatomical sites higher concentrations of azithromycin can overcome the efflux-mediated resistance mechanisms [31]. An alternative explanation is that even though SDSE can be found in different anatomical sites, it is better suited to survive in the vaginal tract [32]. One azithromycin resistant SDSE(A) was isolated in the vaginal tract from a woman included in the azithromycin arm before the intervention was administered. A phylogenetically linked isolate was isolated from the same woman’s VS after the intervention (Additional file 1).

The public health and clinical implications of the selective expansion of SDSE(A) in the vaginal tract are difficult to anticipate. However, similarly to *S. pyogenes,* SDSE can cause invasive disease [33–35]. Lancefield group A SDSE has been described in previous studies from high income settings, including a collection of isolates causing invasive disease in the USA [27]. The SDSE(A) isolated from our study participants have distinct phylogeny to SDSE(A) previously isolated in USA and fall into two distinct clades. In our study, all SDSE(A) isolates harboured *mefA-msrD* genes, whereas both *erm(B)* and *mefA-msrD* genes were found in azithromycin-resistant *S. pyogenes* isolates. While the presence of *mefA* is associated with macrolide resistance, *msrD* has a more dominant role [36, 37]. The presence of both *mefA* and *msrD* may confer high level resistance [37].

The trial was designed to evaluate the effect of intra-partum azithromycin on maternal and neonatal carriage of *S. aureus*, *S. pneumoniae* and GBS that are more prevalent than *S. pyogenes* and therefore the current analysis was underpowered as observed in the comparison of trial arms in the nasopharyngeal swabs, especially in newborns, as carriage of GAS is lower than for those other bacteria. This was an opportunistic study and oropharyngeal rather than nasopharyngeal samples may have been more appropriate for detecting *S. pyogenes* carriage where it would be expected that carriage would have been slightly higher [38]. In any case, the objective of the analysis was to assess the impact of the azithromycin on GAS carriage and there is no reason to believe this should be very different in the oropharynx when compared to the nasopharynx. Overall, our study adds to the growing evidence that GAS may include SDSE as well as *S. pyogenes.*

## Conclusions

In conclusion, this study demonstrates that a simple intervention (single dose of intra-partum oral azithromycin), has the potential to reduce carriage of GAS, an important cause of maternal and neonatal sepsis. The effect of this prophylactic intervention on azithromycin resistant SDSE(A) isolates and its effect on disease needs to be closely monitored when assessing the overall public health potential of prophylactic intra-partum azithromycin.

## Supplementary Information

**Additional file 1**. Whole Genome Sequencing Data.

## Data Availability

All data generated or analysed during this study are included in various published articles and are available from the corresponding author on reasonable request.

## Abbreviations

GAS: Group A *Streptococcus*
WGS: Whole Genome Sequencing
RR: Risk Ratio
SDSE(A): *Streptococcus dysgalactiae* Subspecies *equisimilis* expressing Lancefield group A carbohydrate
